# Several frailty parameters highly prevalent in middle age (50–65) are independent predictors of adverse events

**DOI:** 10.1038/s41598-021-88410-5

**Published:** 2021-04-22

**Authors:** Lauriane Segaux, Amaury Broussier, Nadia Oubaya, Claire Leissing-Desprez, Marie Laurent, Henri Naga, Isabelle Fromentin, Jean-Philippe David, Sylvie Bastuji-Garin

**Affiliations:** 1 Univ Paris Est Creteil, INSERM, IMRB, 94010 Creteil, France; 2 Clinical Research Unit (URC Mondor), AP-HP, Hôpitaux Henri-Mondor, 94010 Creteil Cedex, France; 3 Departments of Geriatric Medicine, AP-HP, Hôpitaux Henri-Mondor, 94010 Creteil, France; 4 Departments of Geriatric Medicine, AP-HP, Hôpitaux Henri-Mondor, 94450 Limeil-Brévannes, France; 5 Department of Public Health, AP-HP, Hôpitaux Henri-Mondor, 94010 Creteil, France

**Keywords:** Health care, Risk factors

## Abstract

Although frailty can arise in middle age, very few studies have investigated frailty before 65 years. Our objectives were to assess the prevalence of frailty parameters in middle-aged individuals and probe the association with future adverse events. We performed cross-sectional and longitudinal analyses of community-dwelling individuals aged 50 to 65 (n = 411, median age: 59.0) having undergone a multidomain geriatric assessment (2010–2015) in an outpatient clinic in the greater Paris area of France (SUCCEED cohort). The primary outcome was a composite measure of adverse events (non-accidental falls, fractures, unplanned hospitalizations, death), recorded in 2016/2017. Multivariable logistic regression models were built to identify independent predictors. Six frailty parameters were highly prevalent (> 20%): low activity (40.1%), exhaustion (31.3%), living alone (28.5%), balance impairment (26.8%), weakness (26.7%), and executive dysfunction (23.2%). Female sex (odds ratio: 2.67 [95% confidence interval: 1.17–6.11]), living alone (2.39 [1.32–4.33]), balance impairment (2.09 [1.16–3.78]), executive dysfunction (2.61, [1.18–5.77]), and exhaustion (2.98 [1.65–5.39]) were independent predictors of adverse events. Many frailty parameters are already altered in middle-aged individuals and are predictive of adverse health events. Our findings highlight a possible need for frailty screening and preventive programs targeting middle-aged individuals.

## Introduction

The concept of frailty was introduced to account for variability in the aging process. This syndrome reflects a decrease in the physiological reserve, and reduces the ability to respond to stress^1^. In older adults, frailty is known to be associated with an increased risk of adverse outcomes, such as falls, fractures, unplanned hospitalizations, and death^2^. Several frailty domains may only be slightly altered, so that early-stage frailty is not necessarily clinically visible^3^.

The most commonly used operational definitions of frailty are based on two different conceptual frameworks. Fried’s rules-based criteria correspond to a physical phenotype^4^, whereas the “Rockwood accumulative model” defines frailty as the accumulation of multiple deficits^5^. These instruments have been studied and validated in populations of individuals aged 65 and over. However, many other measures of frailty have been suggested. According to two consensus papers, an operational definition of frailty should include components from the nutrition, mobility, physical activity, strength, endurance, balance, cognition, senses, mood and social domains^6,7^. Furthermore, frailty can also be found in younger adults^8,9^, and we have reported on frailty profiles in community-dwelling individuals aged 50–75 (median: 61.7)^10^. Moreover, a recent study reported that frailty was associated with an elevated mortality rate among younger adults^8^. We therefore hypothesized that the factors determining the main ageing-related adverse events are already present in middle age.

Although the early detection of frailty is potentially important (since the condition might be reversible in its early stages)^11,12^, the prevalence of various components of frailty assessments (hereafter referred to as “frailty parameters”) among middle-aged populations and the parameters’ relationships with further adverse events have not been extensively documented in the literature. Most of the literature studies have focused on older adults or on a small number of frailty parameters, and none investigated the parameters’ prognostic value in multivariate models^1,8,9,13–21^.

The present study’s objectives were therefore to investigate the prevalence of various frailty parameters in middle-aged community-dwelling individuals (aged 50–65) and assess the parameters’ prognostic value for future adverse health events.

## Methods

### Design and participants

We performed cross-sectional and longitudinal analyses of community-dwelling volunteers aged 50 or over having been prospectively included in the ongoing SUCCEED cohort at an outpatient clinic in a university medical center in the greater Paris area of France^22^. The participants underwent a comprehensive multi-domain geriatric assessment after attending the SUCCessful ageing outpatient Department (SUCCEED) for an initial “prevention and healthy ageing” consultation; they had not been referred by a physician or for a particular health problem. In the present analysis, we selected individuals aged 50–65 and having been recruited between 2010 and 2015. In 2016/2017, the participants were contacted by phone by a geriatrician and asked to provide information on any adverse health events that had occurred since the geriatric assessment.

### Measures

The data were collected prospectively. Performance tests were conducted by trained nurses, and a comprehensive geriatric assessment was performed by a geriatrician. In the present study, we selected components from the comprehensive geriatric assessment’s nutrition, mobility, physical activity, strength, endurance, balance, cognition, senses, mood, and social domains. Self-reported unintentional weight loss over the previous year (regardless of the amount lost) was recorded. Mobility was assessed via a daily pedometer count over a week (low level of physical activity: < 7500 steps per day)^23^, and gait speed over 10 m (slowness: < 1 m/s)^24^. Muscle strength was estimated by the completion time in a five-time sit-to-stand test (≥ 11.19 s)^25^, and maximum dominant-hand grip strength (kg) measured with a dynamometer (JAMAR, Sammons Preston, Bolingbrook, IL, USA). Weakness was defined as grip strength stratified by sex and body mass index^4^. The appendicular lean mass index was estimated using dual-energy X-ray absorptiometry (low muscle mass: < 7.23 kg/m^2^ in men, < 5.67 in women)^26^; sarcopenia was defined as a combination of low muscle mass with low muscle strength (< 30 kg in men and < 20 kg in women) or slowness^26^. The sternal push test and ankle dorsiflexion (< 20°, taken as the cut-off for clinically relevant ankylosis) were used to assess balance. The times in a 10-m walking test with concurrent motor and cognitive tasks were recorded. Overall cognitive performance was evaluated using the Mini-Mental State Examination score adjusted for age and educational level^27^. Episodic memory was assessed using the five-word screening test (< 10/10)^28^. Executive and visuospatial functions were evaluated using the seven-point clock-drawing test (CDT) (< 7/7)^29^, and frontal lobe functions were evaluated using the Frontal Assessment Battery (FAB) (< 16/18)^30^. Mood was explored using the Geriatric Depression Scale (risk of depression: ≥ 11/30, or ≥ 5/15 for the short form)^31^. Hearing impairment was defined as hearing aid use or a poor result in the finger rub test. Living alone was considered to be a proxy for the social domain. Comorbidities were also recorded.

To replicate the Fried phenotype (according to the Cardiovascular Health Study (CHS) criteria)^4^, we considered shrinking (> 4.5 kg in the last year), weakness, exhaustion (a self-reported feeling of general fatigue over the previous year), slowness, and a low level of physical activity (< 7500 steps per day, and no regular physical activity). We classified individuals according to these modified CHS criteria, the number of positive items defined the individuals as frail (≥ 3), pre-frail (1–2) or robust (none).

### Outcome

Due to the small number of adverse events observed during follow-up, we considered a composite outcome variable comprising non-accidental falls (i.e. those not related to sports, DIY or domestic accidents), fractures (hip, spine, and wrist), unplanned hospital admissions, and death. This information was gathered recorded during the 2016/2017 phone interviews with a geriatrician.

### Statistical analysis

Quantitative variables were quoted as the median [interquartile range (IQR)], and qualitative variables as the number (%).

The prevalence of frailty parameters was estimated for the whole population and then (if the overall prevalence was ≥ 5%) in [50–55], [56–60] and [61–65] age groups selected a priori. Potential differences between age classes were tested using a chi-squared test for trend or Cuzick’s trend test, as appropriate. Pairwise comparisons were performed using a chi-squared or Kruskal–Wallis tests; *P* values from multiple pairwise comparisons were corrected using the false discovery rate method^32^.

To investigate frailty parameters that were potentially predictive of adverse events, we used logistic regression models adjusted for the length of follow-up. Given that the number of frail individuals was small, we pooled the pre-frail and frail categories into a "non-robust" phenotype. Odds ratios (ORs) with their 95% confidence intervals (CIs) were estimated for variables with *P* values < 0.15. These variables were then considered in a multivariable logistic regression model. After checking compliance with the missing-at-random hypothesis by exploring the pattern of missingness, we used multiple imputations to maximize the sample size^33^. We assessed the model’s discrimination [area under the receiver-operating characteristic curve (AUC)] and calibration (Hosmer–Lemeshow test). A sensitivity analysis was carried out on complete cases.

We used the same method to assess the relationship between frailty parameters and non-accidental falls.

The threshold for statistical significance was set to *P* < 0.05. Statistical analyses were performed using STATA software (version 15.0, StataCorp, College Station, TX).

The present observational study was reported in accordance with the Strengthening the Reporting of Observational Studies in Epidemiology statement^34^.

### Ethical approval and consent to participate

All participants gave their verbal informed consent prior to inclusion in the study. In line with the French legislation on observational studies, written informed consent was not required. All procedures were performed in accordance with the relevant guidelines and regulations. This study with all procedures was approved by an independent ethics committee (*Comité de Protection des Personnes Ile-de-France X*, Paris, France, reference: 96-2019).

## Results

**Figure 1:**
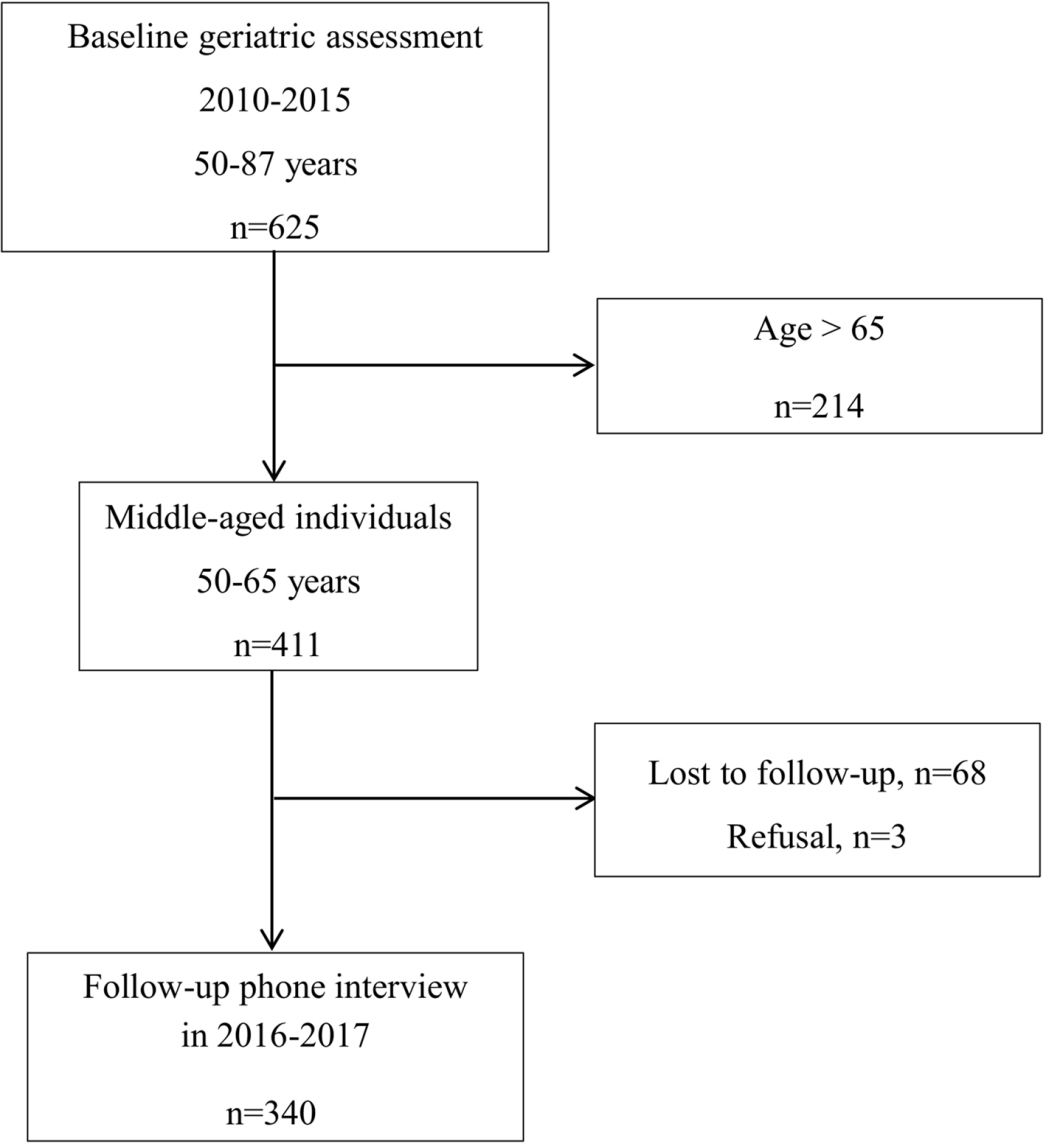
Flow chart for the SUCCEED-04 survey.

Of the 625 individuals included in the SUCCEED survey, the 411 aged ≤ 65 years were analyzed. After a median follow-up of 3 years [2–5], outcomes were available for 340 individuals (82.7%) (Fig. 1).

Table 1 reports on the participants’ baseline characteristics; the median age was 59.0 [55.9–62.1] years, 71.1% were female, and 34.6% were retired. According to the modified physical frailty phenotype, 233 (66.8%) of the participants were pre-frail and 18 (5.2%) were frail.

**Table 1:** Baseline characteristics of the 411 community-dwelling participants.

Characteristics	N (%)
Age in years, median [IQR]	59.0 [55.9–62.1]
Female sex	292 (71.1)
Years of full-time education, median [IQR] (n_missing_ = 8)	14 [11–15]
Retired (n_missing_ = 4)	141 (34.6)
**Comorbidities**	
Number of comorbidities, median [range]	1 [0–5]
Hypertension^a^ (n_missing_ = 3)	89 (21.8)
Diabetes (n_missing_ = 4)	19 (4.7)
Dyslipidemia (n_missing_ = 5)	126 (31.0)
Obesity (body mass index ≥ 30 kg/m^2^) (n_missing_ = 1)	49 (12.0)
Cardiovascular disease^b^ (n_missing_ = 15)	11 (2.8)
Other cardiac diseases^c^ (n_missing_ = 5)	17 (4.2)
Depression (n_missing_ = 3)	58 (14.2)
Cancer (n_missing_ = 4)	19 (4.7)
Thyroid disorders (n_missing_ = 5)	38 (9.4)
Other comorbidities^d^	35 (8.5)

The data are quoted as the number (%), unless otherwise stated; (n_missing_ =) indicates the number of missing data.

*IQR* interquartile range.

^a^Hypertension, defined as a systolic blood pressure ≥ 140 mmHg or a diastolic blood pressure ≥ 90 mmHg or ongoing treatment for hypertension.

^b^Cardiovascular disease includes stroke (n = 6), transient ischemic attack (n = 6) and coronary heart disease (n = 8).

^c^Other cardiac diseases include chronic heart failure (n = 5), valvulopathy (n = 9), or cardiac rhythm disorders (n = 20).

^d^Other comorbidities include: respiratory disorders (asthma, chronic obstructive pulmonary disease and bronchiectasis (n = 29), obstructive sleep apnea syndrome (n = 28), neuropsychological disorders (n = 11), age-related macular degeneration (n = 4), viral hepatitis B or C (n = 4), sickle cell anemia (n = 3), HIV (n = 2), ulcerative colitis (n = 1), and polymyalgia rheumatica (n = 1).

### Prevalence of frailty parameters

Six frailty parameters were highly prevalent (> 20%) in this middle-aged population: living alone, a low level of physical activity, weakness, exhaustion, limited ankle dorsiflexion (a proxy of altered balance), and executive dysfunction (assessed by the CDT).

The number of comorbidities, the time needed to walk ten meters during a concurrent task, and the prevalence of impaired CDT and FAB performance increased significantly with age (Table 2). Gait speed and the prevalence of depressive symptoms decreased significantly with age; a trend towards a decrease was observed for the prevalence of exhaustion (*P* = 0.07). Employment status was associated with both exhaustion and age; hence, after stratification by employment status, exhaustion was no longer associated with age (*P* > 0.47). Lastly, we observed a non-significant trend towards an increase in the prevalence of non-accidental falls in the past year with age (*P* = 0.09) (Table 2).

**Table 2:** Frailty parameters within a population of community-dwellers aged 50–65, by age class (N = 411).

Frailty parameters	Total	Age class			*P* values for trend^a^	*P* values for pairwise comparisons^b^		
		[50–55]	[56–60]	[61–65]		50–55 versus		56–60 versus
	N = 411	(n = 80)	(n = 165)	(n = 166)		56–60	61–65	61–65
Living alone	117 (28.5)	26 (32.5)	40 (24.2)	51 (30.7)	0.82			
Number of comorbidities, median [range]	1 [0–5]	0 [0–2]	1 [0–5]	1 [0–5]	** < 0.001**	**0.02**	** < 0.001**	0.187
**Nutrition**								
Unintentional weight loss in the past year, regardless of amount lost	56 (14.8)	11 (14.1)	25 (16.9)	20 (13.1)	0.69			
Shrinking (unintentional weight loss in the past year > 4.5 kg)	17 (4.4)	–	–	–	–			
**Mobility, muscle strength and activity**								
Number of steps/day < 7500	161 (47.6)	33 (50.8)	61 (44.5)	67 (49.3)	0.91			
Gait speed m/s, median [IQR]	1.43 [1.25–1.67]	1.44 [1.25–1.67]	1.43 [1.25–1.67]	1.43 [1.25–1.54]	**0.02**	0.745	0.057	0.057
Low level of physical activity^c^	150 (40.1)	37 (48.7)	57 (38.3)	56 (37.6)	0.13	0.201	0.201	0.905
Slowness (gait speed < 1 m/s)	6 (1.5)	–	–	–	–			
Completion time in a five-time sit-to-stand test ≥ 11.19 s	33 (8.2)	7 (8.9)	16 (10.1)	10 (6.1)	0.34			
Weakness^d^	109 (26.7)	21 (26.3)	35 (21.6)	53 (31.9)	0.18			
Appendicular lean mass index < 7.23 (males) or < 5.67 (females)	47 (11.4)	9 (11.3)	19 (11.5)	19 (11.5)	0.97			
Sarcopenia	17 (4.2)	–	–	–	–			
**Balance**								
Failure to resist a sternal push	19 (4.8)	–	–	–	–			
Ankle dorsiflexion < 20°	107 (26.8)	22 (28.2)	36 (22.8)	49 (30.1)	0.56			
**Cognition**								
MMSE ≤ lower quartile according to age and educational level	68 (16.9)	13 (16.5)	29 (18.0)	26 (16.1)	0.82			
Five-word test score < 10	10 (2.4)	–	–	–	–			
Seven-point clock-drawing test < 7	95 (23.2)	11 (13.8)	40 (24.5)	44 (26.5)	**0.03**	0.078	0.072	0.683
Frontal assessment battery < 16	51 (12.5)	6 (7.5)	18 (11.0)	27 (16.4)	**0.03**	0.392	0.171	0.233
Time to walk 10 m during a dual task, s, median [IQR]								
Motor dual task	7 [6–8]	7 [6–7.9]	7 [6.1–8]	7 [6.2–8]	**0.03**	0.261	0.09	0.261
Cognitive dual task	7.3 [6.5–8.4]	7 [6–8.2]	7.1 [6.5–8]	7.6 [7–9]	**0.006**	0.368	**0.045**	**0.045**
**Mood**								
Depressive symptoms (GDS ≥ 11/30 or 5/15)	54 (15.3)	15 (23.1)	22 (15.7)	17 (11.6)	**0.03**	0.303	0.093	0.305
Exhaustion	114 (31.3)	27 (39.1)	49 (32.2)	38 (26.6)	0.07	0.317	0.189	0.317
Hearing impairment^e^	66 (16.5)	8 (10.0)	30 (18.9)	28 (17.4)	0.25			
Non-accidental fall(s) in the past year	27 (6.7)	3 (3.8)	9 (5.5)	15 (9.2)	0.09	0.756	0.305	0.305

The data are quoted as the number (%), unless otherwise stated.

*P-*values < 0.05 have been put in bold.

*IQR* interquartile range, *MMSE* mini mental state examination, *GDS* geriatric depression scale.

^a^*P* value for trend from the chi-squared statistic or Cuzick's test.

^b^*P* value for the chi-squared or Kruskal–Wallis tests, corrected for multiple comparisons using the false-discovery rate method, for variables that yielded P values < 0.15.

^c^Low level of physical activity: no regular physical activity (walking, recreational sports, and other physical activities) and number of steps/day < 7500.

^d^Weakness was defined as grip strength (kg), stratified by sex and body mass index.

^e^Abnormal finger rub test or use of a hearing aid.

In pairwise comparisons, the number of comorbidities was significantly higher in the 56–60 group (relative to the 50–55 group), and significantly lower gait speed (during normal walking and during a cognitive dual task) was observed from the age of 61 onwards (Table 2).

### Association between frailty parameters and adverse events

The following adverse outcomes were reported by 68 of the 340 middle-aged subjects (20%) with follow-up data: deaths (N = 2, 0.6%), non-accidental falls (N = 47, 14.0%), fractures (N = 3, 0.9%), and unplanned hospitalizations (N = 21, 6.3%).

We found that female sex, living alone, weakness, altered balance, abnormal FAB score, and exhaustion were significant predictors of adverse events in the univariable analysis (Table 3).

**Table 3:** Frailty parameters associated with the occurrence of adverse events among community-dwelling individuals aged 50–65 years.

Characteristics	Composite outcome^a^		OR [95% CI]^b^	*P-*value^b^
	No event N = 272	At least one event N = 68		
Age, year^c^	59 [56–62]	59 [56–62]		0.87
Sex (female)	184 (67.6)	60 (88.2)	3.64 [1.66–7.95]	**0.001**
Education, year^c^	14 [11–15]	12 [9.5–15]	0.93 [0.85–1.01]	0.09
Living alone	62 (22.8)	31 (45.6)	3.00 [1.71–5.26]	** < 0.001**
Retired	96 (35.6)	24 (35.3)		0.90
Number of comorbidities^c^	1 [0–1]	1 [0–1.5]		0.49
**Nutrition**				
Unintentional weight loss in the past year				
Regardless of amount lost	38 (15.2)	5 (7.8)		0.21
> 4.5 kg (Shrinking)	14 (5.5)	2 (3.1)		0.53
**Mobility, muscle strength and activity**				
Number of steps/day < 7500	109 (47.4)	24 (45.3)		0.75
Gait speed m/s^c^	1.4 [1.3–1.7]	1.4 [1.3–1.7]		0.68
Low level of physical activity^d^	97 (38.2)	22 (36.7)		0.94
Slowness (gait speed < 1 m/s)	3 (1.1)	2 (2.9)		0.30
Appendicular lean mass index < 7.23 (males) or < 5.67 (females)	30 (11.0)	10 (14.7)		0.33
Completion time in a five-time sit-to-stand test ≥ 11.19 s	18 (6.8)	7 (10.6)		0.37
Weakness^e^	71 (26.2)	26 (39.4)	1.82 [1.04–3.21]	**0.04**
Sarcopenia	11 (4.0)	5 (7.5)		0.20
**Balance**				
Failure to resist a sternal push	15 (5.7)	2 (3.0)		0.35
Ankle dorsiflexion < 20°	66 (25.2)	29 (43.3)	2.21 [1.26–3.87]	**0.006**
**Cognition**				
MMSE ≤ lower quartile according to age and educational level	45 (16.9)	6 (9.0)		0.15
Five-word test score < 10	7 (2.6)	1 (1.5)		0.54
Seven-point clock-drawing test < 7	59 (21.7)	18 (27.3)		0.27
Frontal assessment battery < 16	27 (10.0)	15 (22.4)	2.54 [1.26–5.13]	**0.009**
Time to walk 10 m during a dual task, s^c^				
Motor dual task	7 [6–8]	7 [6.5–8]		0.64
Cognitive dual task	7.2 [6.5–8]	7.8 [6.5–9]		0.86
**Mood**				
Depressive symptoms (GDS ≥ 11/30 or 5/15)	31 (13.1)	10 (18.9)		0.24
Exhaustion	67 (27.3)	29 (46.8)	2.50 [1.40–4.48]	**0.002**
Non-accidental fall(s) in the past year	15 (5.6)	6 (9.0)		0.27
Non-robust according to the modified CHS criteria^f^	183 (68.5)	53 (77.9)	1.72 [0.89–3.32]	0.10
Hearing impairment^g^	38 (14.5)	11 (17.2)		0.38

The data are quoted as the number (%), unless otherwise stated. Abbreviations: OR, odds ratio; CI, confidence interval; MMSE, Mini Mental State Examination; GDS, Geriatric Depression Scale; CHS, Cardiovascular Health Study.

*P-*values < 0.05 have been put in bold.

^a^The composite outcome comprised the following adverse events: non-accidental falls (those not related to sports, DIY, or domestic accidents), fractures (hip, spine, and wrist), unplanned hospital admissions, and death. Some patients had multiple events.

^b^Logistic regression analyses were adjusted for the length of follow-up.

^c^Reported as the median [interquartile range].

^d^Low level of physical activity: no regular physical activity (walking, recreational sports, and other physical activities) and number of steps/day < 7500.

^e^Weakness was defined as grip strength (kg), stratified by sex and body mass index.

^f^The modified CHS criteria were shrinking, self-reported exhaustion, weakness, slowness, and low physical activity (< 7500 steps/day and no regular physical activity). The number of positive items defined the individuals as frail (≥ 3), pre-frail (1–2) or robust (none); pre-frail and frail categories were pooled for the analysis.

^g^Abnormal finger rub test or use of a hearing aid.

Non-significant trends (*P* ≤ 0.10) were observed for a lower educational level and a non-robust modified Fried phenotype.

In a multivariable analysis with multiple imputations, female sex, living alone, exhaustion, altered balance and a FAB score < 16 were independent predictors of subsequent adverse events (Table 4).

**Table 4:** Multivariable logistic regression analyses of frailty parameters predicting the occurrence of adverse health events (N = 402).

	Composite outcome^a^		Non-accidental falls	
	Adjusted OR [95% CI]	*p*-value	Adjusted OR [95% CI]	*p*-value
Female sex	2.67 [1.17–6.11]	0.02	8.07 [1.74–37.5]	0.008
Living alone	2.39 [1.32–4.33]	0.004	2.39 [1.22–4.69]	0.01
Balance impairment (ankle dorsiflexion < 20°)	2.09 [1.16–3.78]	0.02	1.80 [0.91–3.57]	0.09
Executive function impairment (FAB score < 16)	2.61 [1.18–5.77]	0.02	2.48 [1.02–6.06]	0.046
Exhaustion	2.98 [1.65–5.39]	< 0.001	2.32 [1.15–4.68]	0.02

*OR* odds ratio, *CI* confidence interval, *FAB* frontal assessment battery.

^a^The composite outcome comprised the following adverse events: non-accidental falls (those not related to sports, DIY, or domestic accidents), fractures (hip, spine, and wrist), unplanned hospital admissions, and death. Some patients had multiple events.

Multivariable models (after multiple imputation for missing data) were also adjusted for length of follow-up.

Similar results were found in the sensitivity analyses of complete cases (Supplementary Table 1). The model had good discriminative power (AUC = 0.76 [0.69–0.82]) and was well calibrated (*P* = 0.39). Similar results were observed when considering non-accidental falls as an outcome (Table 4 and Supplementary Table 2).

## Discussion

In a population of middle-aged community-dwellers (aged 50–65 years), three of the modified CHS criteria (exhaustion, weakness, and a low level of physical activity) and three other frailty parameters (living alone, altered balance, and executive dysfunction) were already highly prevalent (> 20%). Four of these parameters (namely living alone, exhaustion, altered balance, and executive dysfunction) and female sex were independently associated with subsequent adverse events—suggesting the need to screen for frailty among middle-aged adults, with a view to including them in targeted interventional programs. Lastly, gait speed and executive function worsened significantly with age.

To the best of our knowledge, the present study is the first to have (i) estimated the prevalence of a large number of frailty parameters among middle-aged community-dwellers (aged 50–65) and (ii) evidenced associations with adverse events. Most previous studies focused on older (over-65) participants or considered a small number of criteria (either the five CHS criteria or a few specific parameters), and none investigated the parameters’ prognostic value in multivariate models^8,9,13–21^. Although the frailty parameters were associated with adverse health events, the parameters’ clinical significance among younger adults may differ from that among older adults. Indeed, the concept of frailty should be applied to younger adults with caution. Moreover, we cannot say that a change in these parameters reflects a decrease in functional reserves. However, our findings indicate that these frailty components should be assessed in middle age.

Although the observed prevalences of exhaustion, a low level of physical activity and living alone, were in line with previous reports^9,20,35–37^, the prevalence of weakness was higher than expected^8,9,20^. Our finding of similar prevalence of weakness and altered balance (26.7% and 26.8%, respectively) is consistent with the literature data on the link between these two frailty parameters in older women^38^; however, the underlying mechanisms have yet to be elucidated. To the best of our knowledge, the prevalence of balance impairment among the middle-aged adults has not previously been reported. However, a deterioration in balance among young and middle-aged individuals and then further deterioration after the age of 60 has been reported^39^. Although the community-dwellers included in the SUCCEED cohort were not expected to have dementia, a substantial proportion (23.2%) displayed impairments in executive function. In line with a previous report, we also observed an age-related decline in executive and visuospatial functions^40^ but not in global cognitive performance. Indeed, normal ageing appears to be associated with selective changes in certain specific cognitive domains coordinated by the prefrontal area (e.g. executive function), rather than with overall cognitive decline^41^.

In line with the literature data, we observed a gradual increase with age in reduced speed and a decrease in depressive symptoms^13,35,42^. Although exhaustion is one of the CHS criteria, we observed a trend towards a decrease in its prevalence with age—in line with some previous reports^8,20^. However, our results suggest that employment status (i.e. being retired or not) is a confounding factor in this association.

Interestingly, the five independent predictors of adverse events in middle-aged community-dwellers reported here (female sex, living alone, exhaustion, altered balance, and executive function impairment) are well-known risk factors for adverse events in persons 65 years or older^14–16,43^. In line with our results, a few studies have documented an increased risk of falls, fractures or unplanned hospitalizations associated with female sex, living alone, and altered balance in under-65 community-dwellers^13,17,18^. However, none of these studies reported on all these parameters in a multivariable model. The present study is the first to have observed an association between the risk of adverse events and executive dysfunction in healthy middle-aged adults. Although an earlier study showed that executive dysfunction is a likely precursor of frailty and disability in older adults, our results suggest that executive function impairment is also important in apparently healthy middle-aged individuals^44^.

Loneliness and social frailty are known predictors of physical frailty among individuals 65 years or older^45,46^. In our middle-aged population, participants living alone (a proxy of social frailty) were more likely to experience an adverse event than participants not living alone. Thus, these individuals may be potential target for frailty prevention.

Very few studies have investigated the relationships between the Fried phenotype and adverse events in healthy middle-aged populations^8^. The small number of participants with a frail physical phenotype (according to the modified CHS criteria) prevented us from assessing whether the modified phenotype predicted adverse events. However, exhaustion was the only modified CHS criterion that predicted adverse events in our study. Our results therefore suggest that although it might be useful to detect frailty parameters among middle-aged individuals, focusing solely on the physical frailty criteria may not be advisable. Hence, we recommend screening for alterations in balance, executive functions and exhaustion in middle age—especially among people living alone and women. Once our present findings have been validated in another setting, a screening tool that takes account of the impaired domains could be developed.

### Clinical implications

Although the effects were unclear and inconsistent, several studies have shown that interventions based on cognitive training and nutritional interventions or physical exercise are effective in preventing pre-frailty or slowing frailty progression in older adults^11,12^. Given that the middle-aged individuals studied here already had a high prevalence of frailty parameters and that some of the latter were associated with adverse events, our results emphasize the need for targeted actions in a younger-than-usual age group.

### Strengths and weaknesses

We prospectively recorded frailty parameters from almost all domains of geriatric assessment using validated scales.

The recruitment of participants wishing to benefit from a comprehensive geriatric assessment at a single center may limit the external validity of our results. However, since the prevalences of many of the frailty parameters observed here were similar to those reported previously, major selection bias is unlikely to have been present^9,13,20,35–37^. Furthermore, the use of a multiple imputation procedure enabled us to consider participants who were lost to follow-up and thus to limit potential selection bias. The fact that a sensitivity analysis of complete cases produced similar results suggests that our findings are robust.

We compared prevalence rates across age groups, rather than changes over time in frailty parameters among the same population of individuals; hence, we cannot rule out a potential generational effect. The recording of adverse events through phone interviews constitutes a further study limitation.

## Conclusion

In a population of middle-aged community-dwellers aged 50–65, we observed a high prevalence of frailty parameters known to be associated with adverse events in older people. Furthermore, we found that parameters from various frailty domains (living alone, exhaustion, balance impairment, and executive dysfunction) and female sex were independent predictors of adverse events. Our findings highlight a possible need for frailty screening and preventive programs that target middle-aged adults.

## Supplementary Information

Supplementary Information.

## Data Availability

The datasets analyzed during the current study are not publicly available because they are the property of Assistance Publique Hôpitaux de Paris. Any individual may apply for data by contacting the Direction de la Recherche Clinique et de l’Innovation (DRCI) at drc.secretariat-promotion@aphp.fr.

## Abbreviations

AUC: Area under the receiver-operating characteristic curve
BMI: Body mass index
CDT: Seven-point clock-drawing test
CI: Confidence interval
CHS: Cardiovascular health study
FAB: Frontal assessment battery
GDS: Geriatric depression scale
IQR: Interquartile range
MMSE: Mini mental state examination
OR: Odds ratio
