# Supplementary material for: Several frailty parameters highly prevalent in middle age (50–65) are independent predictors of adverse events
Source: Sci Rep. 2021 Apr 22;11:8774. doi: 10.1038/s41598-021-88410-5 (PMC8062562; doi:10.1038/s41598-021-88410-5)
Supplement: Supplementary file 1 — Supplementary Information. [file 41598_2021_88410_MOESM1_ESM.docx]

**Several frailty parameters highly prevalent in middle age (50-65) are independent predictors of adverse events**

Lauriane Segaux^1,2*^, Amaury Broussier^1,3║^, Nadia Oubaya^1,4║^, Claire Leissing-Desprez^1,3^, Marie Laurent^1,3^, Henri Naga^3^, Isabelle Fromentin^3^, Jean-Philippe David^1,3*^, Sylvie Bastuji-Garin^1,2,4*^

^1^Univ Paris Est Creteil, INSERM, IMRB, F-94010 Creteil, France

^2^AP-HP, Hôpitaux Henri-Mondor, Clinical Research Unit (URC Mondor), F-94010 Creteil, France

^3^AP-HP, Hôpitaux Henri-Mondor, Departments of Geriatric Medicine, F-94010 Creteil and F-94450 Limeil-Brévannes, France

^4^AP-HP, Hôpitaux Henri-Mondor, Department of Public Health, F-94010 Creteil, France

^*^ Corresponding author

^║^These authors contributed equally to the study.

| **Supplementary Table 1.** Multivariable logistic regression analyses of frailty parameters predicting the occurrence of adverse health events (sensitivity analyses of complete cases, N=292). | | | | | | | |
| --- | --- | --- | --- | --- | --- | --- | --- |
|  | **Adjusted OR**  **[95% CI]** | | ***P*-value** |  |  | |  |
| Female sex | 3.1 | [1.28-6.92] | **0.01** |  |  |  |  |
| Living alone | 2.86 | [1.51-5.44] | **0.001** |  |  |  |  |
| Balance impairment (ankle dorsiflexion <20°) | 2.36 | [1.23-4.51] | **0.009** |  |  |  |  |
| Executive function impairment (FAB score <16) | 2.13 | [0.92-4.93] | 0.08 |  |  |  |  |
| Exhaustion | 2.54 | [1.34-4.81] | **0.004** |  | - | | |
|  | - | | |  |  |  |  |
| Abbreviations: OR, odds ratio; CI, confidence interval; FAB, Frontal Assessment Battery | | | | | | | |
| ^a^ The multivariable model was also adjusted for the length of follow-up | | | | | | | |
|  | | | | | | | |

| **Supplementary Table 2.** Frailty parameters associated with the occurrence of non-accidental falls among community-dwelling individuals aged 50-65 years (univariate analyses)**.** | | | | | | |
| --- | --- | --- | --- | --- | --- | --- |
|  | **Non-accidental falls ^a^** | |  |  | |  |
| **Characteristics** | **No** | **Yes** | **OR [95%CI] ^b^** | ***P-*value^b^** | |  |
|  | N=292 | N=47 |  |  |  |  |
| Age, years^c^ | 59 [56-62] | 59 [56-62] |  | 0.74 | |  |
| Sex (female) | 198 (67.8) | 45 (95.7) | 10.71 [2.54-45.09] | **0.001** | |  |
| Education, years^c^ | 14 [11-15] | 12 [9-15] | 0.93 [0.84-1.03] | 0.16 | |  |
| Retired | 103 (35.5) | 16 (34.0) |  | 0.84 | |  |
| Living alone | 70 (24.0) | 23 (48.9) | 3.07 [1.63-5.79] | **<0.001** | |  |
| **Number of comorbidities**^c^ | 1 [0-1] | 1 [0-2] |  | 0.19 | |  |
| **Nutrition** |  | |  |  | |  |
| Unintentional weight loss in the past year |  |  |  |  | |  |
| Regardless of amount lost | 39 (14.6) | 4 (8.9) |  | 0.34 | |  |
| >4.5 kg (Shrinking) | 14 (5.1) | 2 (4.4) |  | 0.87 | |  |
| **Mobility, muscle strength and activity** |  | |  |  | |  |
| Number of steps/day <7500 | 115 (46.7) | 18 (48.6) |  | 0.82 | |  |
| Gait speed m/s^c^ | 1.4 [1.3-1.7] | 1.4 [1.2-1.6] | 0.41 [0.13-1.26] | 0.12 | |  |
| Low level of physical activity ^d^ | 103 (37.9) | 16 (38.1) |  | 0.98 | |  |
| Slowness (gait speed <1 m/s) | 3 (1.0) | 2 (4.3) | 4.27 [0.69-26.32] | 0.12 | |  |
| Appendicular lean mass index <7.23 (males) or <5.67 (females) | 31 (10.6) | 8 (17.0) |  | 0.20 | |  |
| Completion time in a five-time sit-to-stand test ≥11.19 s | 18 (6.3) | 6 (13.0) | 2.21 [0.82-5.94] | 0.12 | |  |
| Weakness^e^ | 77 (26.6) | 19 (41.3) | 1.95 [1.02-3.70] | **0.04** | |  |
| Sarcopenia | 12 (4.1) | 4 (8.5) |  | 0.19 | |  |
| **Balance** |  | |  |  | |  |
| Failure to resist a sternal push | 16 (5.7) | 1 (2.2) |  | 0.34 | |  |
| Ankle dorsiflexion <20° | 75 (26.6) | 19 (41.3) | 1.95 [1.02-3.72] | **0.04** | |  |
| **Cognition** |  | |  |  | |  |
| MMSE ≤ lower quartile according to age and educational level | 45 (15.7) | 5 (10.9) |  | 0.40 | |  |
| Five-word test score <10 | 7 (2.4) | 1 (2.2) |  | 0.93 | |  |
| Seven-point clock-drawing test <7 | 66 (22.6) | 11 (24.4) |  | 0.79 | |  |
| Frontal Assessment Battery <16 | 32 (11.0) | 10 (21.7) | 2.26 [1.02-5.00] | **0.04** | |  |
| Time to walk 10 meters during a dual task, s^c^ |  |  |  |  | |  |
| Motor dual task | 7 [6-8] | 7 [6.5-8] |  | 0.80 | |  |
| Cognitive dual task | 7.2 [6.5-8] | 7.9 [6.5-9] |  | 0.84 | |  |
| **Mood** |  | |  |  | |  |
| Depressive symptoms (GDS ≥11/30 or 5/15) | 32 (12.7) | 8 (20.5) |  | | 0.20 |  |
| **Exhaustion** | 75 (28.6) | 20 (46.5) | 2.23 [1.15-4.33] | **0.02** | |  |
| **Non-accidental fall(s) in the past year** | 16 (5.6) | 5 (10.6) |  | 0.19 | |  |
| **Non-robust according to the modified CHS criteria^f^** | 202 (69.7) | 39 (83.0) | 1.80 [0.79-4.10] | 0.16 | |  |
| **Hearing impairment^g^** | 39 (13.9) | 10 (22.7) | 1.91 [0.86-4.25] | 0.11 | |  |
| Note. The data are quoted as the number (%), unless otherwise stated. Abbreviations: OR, odds ratio; CI, confidence interval; MMSE, Mini Mental State Examination; GDS, Geriatric Depression Scale; CHS, Cardiovascular Health Study | | | | | | |
| ^a^ Non-accidental falls were those not related to sports, DIY, or domestic accidents; ^b^Logistic regression analyses were adjusted for the length of follow-up; ^c^Reported as the median [interquartile range]; ^d^Low level of physical activity: no regular physical activity (walking, recreational sports, and other physical activities) and number of steps/day<7500; ^e^ Weakness was defined as grip strength (kg), stratified by gender and body mass index; ^f^The modified CHS criteria were shrinking, self-reported exhaustion, weakness, slowness, and a low level of physical activity. The number of positive items defined the individuals as frail (≥3), pre-frail (1-2) or robust (none); pre-frail and frail categories were pooled for the analysis; ^g^Abnormal finger rub test or use of a hearing aid | | | | | | |
